# Effect of age as a continuous variable in early-stage endometrial carcinoma: a multi-institutional analysis in China

**DOI:** 10.18632/aging.203367

**Published:** 2021-08-09

**Authors:** Shuai Sun, Lijuan Zou, Tiejun Wang, Zi Liu, Jianli He, Xiaoge Sun, Wei Zhong, Fengju Zhao, Xiaomei Li, Sha Li, Hong Zhu, Zhanshu Ma, Wenhui Wang, Meng Jin, Fuquan Zhang, Xiaorong Hou, Lichun Wei, Ke Hu

**Affiliations:** 1 Department of Radiation Oncology, Peking Union Medical College Hospital, Chinese Academy of Medical Sciences and Peking Union Medical College, Beijing, P.R. China; 2 Department of Radiation Oncology, Xijing Hospital, Air Force Medical University of PLA (The Fourth Military Medical University), Xi’an, P.R. China; 3 Department of Radiation Oncology, The Second Hospital of Dalian Medical University, Dalian, P.R. China; 4 Department of Radiation Oncology, The Second Hospital Affiliated by Jilin University, Changchun, P.R. China; 5 Department of Radiation Oncology, First Affiliated Hospital of Xi’an Jiaotong University, Xi’an, P.R. China; 6 Department of Radiation Oncology, The General Hospital of Ningxia Medical University, Ningxia, P.R. China; 7 Department of Radiation Oncology, The Affiliated Hospital of Inner Mongolia Medical University, Inner Mongolia, P.R. China; 8 Gynaecological Oncology Radiotherapy, Affiliated Tumor Hospital, Xinjiang Medical University, Urumqi, P.R. China; 9 Department of Radiation Oncology, Gansu Provincial Cancer Hospital, Lanzhou, Gansu, P.R. China; 10 Department of Radiation Oncology, Peking University First Hospital, Beijing, P.R. China; 11 Department of Radiation Oncology, The 940th Hospital of Joint Logistics Support Force of Chinese People's Liberation Army, Lanzhou, Gansu, P.R. China; 12 Department of Radiation Oncology, Xiangya Hospital Central South University, Hunan, P.R. China; 13 Department of Radiation Oncology, Affiliated Hospital of Chi feng University, Inner Mongolia, P.R. China; 14 Department of Radiation Oncology, The First Affiliated Hospital of Sun Yat-Sen University, Guangzhou, Guangdong, P.R. China

**Keywords:** endometrial carcinoma, age, adjuvant radiotherapy, cancer-specific survival

## Abstract

Objective: To explore the effect of age at diagnosis as a continuous variable on survival and treatment choice of patients with early-stage endometrial carcinoma (EC).

Materials and Methods: We retrospectively analyzed data from patients with early-stage EC from January 1999 to December 2015 in multiple institutions in China. All patients received primary hysterectomy/bilateral salpingo-oophorectomy and adjuvant radiotherapy for EC confirmed pathology of stage I and II disease (FIGO 2009 staging). All patients were divided into low-risk, intermediate-risk, high-intermediate-risk and high-risk groups according to ESMO-ESGO-ESTRO risk classification.

Results: The median follow-up time was 57months, and the 5-year cancer-specific survival (CSS) was 95.7%. Age as a continuous variable was an independent prognostic factor for CSS. With an increase in age, the hazard ratio (HR) for CSS increases gradually. Other independent prognostic factors included myometrial invasion (MI), grade, and chemotherapy. In the stratified analysis of age, the HRs of age on CSS in patients >70y were 5.516, 5.015, 4.469, 4.618, 5.334, and 5.821 after adjusting for cancer characteristics, local treatment, chemotherapy and treatment-related late toxicity. In patients 66-70-year-old, the HRs were 2.509, 2.074, 2.101, 2.091, 2.157 and 1.621 after adjusting for the above covariates. In patients ≤65y, there was no significant difference in the HR of age on CSS after adjustment.

Conclusion: Age as a continuous variable is an independent prognostic factor and 65 year-old may be the best cut-off point for CSS in patients with early-stage EC in the Asian population. Quality of life should be given greater weight in the choice of therapeutic schedule for those patients >70 y.

## INTRODUCTION

Endometrial carcinoma (EC) is the most common gynecologic malignancy in developed countries. In China, EC has become the second-most common gynecological cancer, with approximately 68,900 cases diagnosed and 16,000 related deaths in 2015 [1]. In the United States, its incidence is steadily increasing with approximately 61 880 new cases diagnosed and 12 160 related deaths in 2019 [2]. Adult women of all ages can suffer from the disease; however, more than 90% of EC occurs in women over 50y. Most ECs are in the early stage (stage I and II) at the time of diagnosis with favorable survival outcomes [3]. Usually, surgery is the primary treatment for early-stage EC. Postoperative treatment, such as radiotherapy and/or chemotherapy, is added according to individual risk factors to reduce the risks of recurrence and to achieve a better prognosis [4]. In many studies, older age at the time of diagnosis of EC is associated with poor prognosis [4–11]. NCCN guidelines also describe age ≥60y as one of the potential adverse risk factors. However, most studies analyzed age as a categorical variable, resulting in a large amount of prognostic information being lost because of the dichotomization of age. Therefore, we aimed to explore the relationship between age at diagnosis and prognosis and whether it is independent of cancer characteristics, initial treatment and treatment-related late toxicity. In this paper, age as a continuous variable was included in the Cox model to analyze its impact on the survival and treatment choice of patients with early-stage EC.

## RESULTS

### Clinical characteristics

A total of 1024 patients with a median age of 57 years (range 23-86 y) were included in the study. The median follow-up time was 57 months (3-237 months). Eight hundred ninety-four patients (87.3%) were staged as I (FIGO2009), and 130 patients (12.7%) were staged as II. A total of 681cases (66.5%) underwent completely staged surgery. The median time interval between surgery and radiotherapy was 38 days (14-238 days). The median number of lymph node dissections was 17 (range 4-65). Two hundred eleven patients (20.6%) underwent chemotherapy. The clinical characteristics and initial treatment of the patients are shown in Table 1.

**Table 1:** Clinical characteristics and initial treatment of all 1024 patients.

**Variables**		**All patients(%)**
Surgical type	Completely surgically staged	681(66.5%)
	Incompletely surgically staged	343(33.5%)
Pathology	endometrioid carcinoma	966(94.3%)
	Non endometrioid carcinoma	58(5.7%)
Grade^a^	Grade1	334(32.6%)
	Grade2	444(43.4%)
	Grade3	246(24.1%)
MI	≥1/2	491(47.9%)
	<1/2	533(52.1%)
LVSI	Positive	157(15.3%)
	Negative	867(84.7%)
LUSI	Yes	293(28.6%)
	No	731(71.4%)
CSI	Yes	130(12.7%)
	No	894(87.3%)
FIGO 2009 stage	Ia	473(46.2%)
	Ib	421(41.1%)
	II	130(12.7%)
ESMO-ESGO-ESTRO Risk classification	Low risk	291(28.4%)
	Intermediate risk	298(29.1%)
	High intermediate risk	180(17.6%)
	High risk	255(24.9%)
Radiotherapy mode	EBRT alone	136(13.3%)
	VBT alone	475(46.4%)
	EBRT+VBT	413(40.3%)
chemotherapy	Yes	211(20.6%)
	No	813(79.4%)

^a^Endometrioid cancers were designated as grade 1, 2, or 3. All poorly differentiated cancers, uterine papillary serous, and clear cell cancers were designated as grade 3, LUSI, lower uterine segment invasion; LVSI, lymphatic vascular space invasion; EBRT, external beam radiotherapy; VBT, vaginal brachytherapy; MI, myometrial invasion; CSI, cervical stromal invasion.

In patients receiving EBRT, 37.8% received CRT, 21.8% received 3DCRT and 40.3% received IMRT. The dose range to EBRT was 40-50.4Gy in 25-28 fractions. When VBT is used as a boost to EBRT, the dose to the vaginal mucosa was 8-25Gy in 2-5 fractions. For postoperative VBT alone, the dose to the vaginal mucosa was 25-40Gy in 5-8 fractions.

### Survival and toxicity analysis

The 5-year CSS was 95.7%. Thirty-eight patients died of EC, of whom 36 had distant metastasis. In the first disease progression, 2 cases had local regional recurrence alone, 6 cases had both distant metastasis and local regional recurrence and 30 cases had distant metastasis. The metastatic sites included peritoneal dissemination, lung, liver, bone, brain and lymph node metastasis.

The incidence of Grade 2 and above late rectal, urinary and hematologic toxicity was 3.9%, 0.7% and 1.3%, respectively. The incidence rates of late rectal and urinary toxicities Grade≥2 were related to EBRT±VBT (P=0.023 and 0.035). The incidence rate of late hematologic toxicities Grade≥2 was not related to chemotherapy or EBRT±VBT (P=0.1 and 0.142) (Table 2). The incidence of Grade 3 and above late rectal, urinary and hematologic toxicity was 0.2%, 0% and 0.2%, respectively. The incidence rate of late toxicities Grade≥3 was not related to EBRT±VBT, VBT or chemotherapy (P >0.05). No patients died of treatment-related toxicity.

**Table 2:** Incidence of treatment-related late toxicity Grade≥2 in each treatment group.

	**EBRT±VBT**			**VBT alone**			**Chemotherapy**		
	**Yes**	**No**	**P**	**Yes**	**No**	**P**	**Yes**	**No**	**P**
Late rectal toxicity Grade≥2	5.3%	2.2%	0.023	2.2%	5.3%	0.023	3.8%	3.9%	1
Late urinary toxicity Grade≥2	1.2%	0	0.035	0	1.2%	0.035	1.1%	0.6%	0.610
Late hematologic toxicity Grade≥2	1.4%	1.2%	1	1.2%	1.4%	1	2.7%	1.0%	0.142

### Analysis of prognostic factors

**Figure 1:**
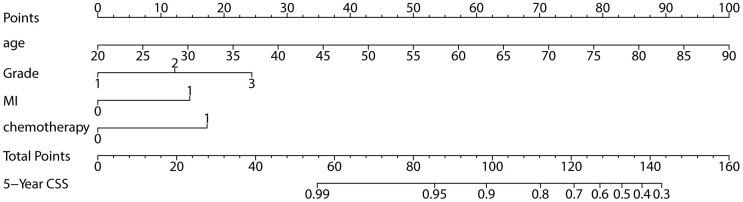
Nomogram for 5-year CSS.

Age as a continuous variable was used in the multivariate analysis. The multivariate analysis showed that age, grade, MI and chemotherapy were independent prognostic factors for CSS (Table 3). The nomogram is shown in Figure 1. Older age, higher grade, MI≥1/2 and chemotherapy were high-risk factors for cancer-specific death.

**Table 3:** Results of the multivariate analysis of all 1024 patients for CSS.

Variable	HR	P	95% CI	
			2.5%	97.5%
Age	1.08	<0.0001	1.042	1.124
Grade	1.59	0.0163	1.089	2.320
MI	2.25	0.0243	1.111	4.541
Chemotherapy	2.42	0.0212	1.141	5.146

CSS, cancer-specific survival; HR, hazard ratio; CI, confidence interval.

**Figure 2:**
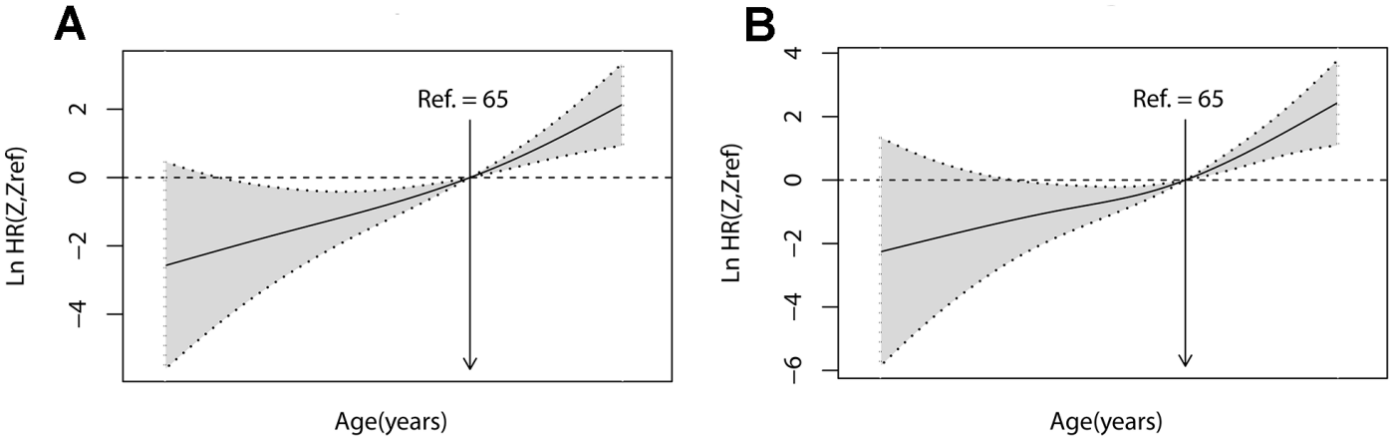
**Linear-dependent effect of increasing age on CSS.** The estimated logarithm HRs (lnHR, solid line) with 95% CIs (shading) for the association of patient age with CSS in 1024 patients based on the dfmacoxas smooth HR - the optimal extended Cox-type additive hazard regression unadjusted model (**A**) or the model adjusted for grade, MI, and chemotherapy (**B**). dfmacox=degrees of freedom in multivariate additive Cox models.

Therefore, age as a continuous variable for CSS is an independent prognostic factor, accounting for a large proportion of the prognostic risk. To quantify the prognostic effect of age, the patient's age was used as a continuous variable, and P-spline was used to enter Cox proportional hazards regression in smooth HR. The results showed that the risk (lnHR) of mortality increased steadily with age (Figure 2A). After adjusting for covariates (grade, MI, and chemotherapy), similar trends were found in the risk of death (lnHR) (Figure 2B).

An age cut-off value of 65 years, defined by ROC analysis, was used as the reference value for calculating the HR. Therefore, the patients were divided into four age groups: ≤ 50 y, 51-65 y, 66-70 y and>70 y. The clinical characteristics of each group are shown in Table 4. With an increase in age, there were fewer completely surgically staged operation, more deep MIs, more VBT alone, fewer chemotherapy received, more treatment-related late toxicity Grade≥2 and cancer-specific mortality.

**Table 4:** Characteristics at baseline for 1024 patients with EC stratified by age.

**Characteristic**		**All patients** **1024(%)**	**Age groups (years)**				**P**
			**≤50**	**51-65**	**66-70**	**>70**	
			**227(%)**	**675(%)**	**65(%)**	**57(%)**	
Completely surgically staged		681(66.5%)	156(68.7%)	464(68.8%)	37(57.1%)	24(42.1%)	0.001
Endometrioid carcinoma		966(94.3%)	218(96.0%)	635(94.1%)	58(89.2%)	55(96.5%)	0.173
Grade	G1	334(32.6%)	85(37.4%)	219(32.4%)	17(26.2%)	13(22.8%)	0.230
	G2	444(43.4%)	96(42.3%)	293(43.4%)	28(43.1%)	27(47.4%)	
	G3	246(24.58%)	46(20.3%)	163(24.1%)	20(30.8%)	17(29.8%)	
MI≥1/2		491(47.9%)	71(31.3%)	345(51.1%)	40(61.5%)	35(61.4%)	0
LVSI+		157(15.3%)	29(12.8%)	110(16.3%)	11(16.9%)	7(12.3%)	0.539
LUSI+		293(28.6%)	94(41.4%)	175(25.9%)	9(13.8%)	15(26.3%)	0
CSI+		130(12.7%)	47(20.7%)	70(10.4%)	8(12.3%)	5(8.8%)	0.001
FIGO 2009 stage	Ia	474(46.3%)	129(56.8%)	302(44.7%)	22(33.8%)	21(36.8%)	0
	Ib	421(41.1%)	51(22.5%)	304(45.0%)	35(53.8%)	31(54.4%)	
	II	129(12.6%)	47(20.7%)	69(10.2%)	8(12.3%)	5(8.8%)	
ESMO-ESGO-ESTRO Risk classification	Low risk	291(28.4%)	90(39.6%)	174(25.8%)	11(16.9%)	16(28.1%)	0
	Intermediate risk	298(29.1%)	40(17.6%)	217(32.1%)	21(32.3%)	20(35.1%)	
	High-intermediate risk	180(17.6%)	34(15.0%)	125(18.5%)	15(23.1%)	6(10.5%)	
	High risk	255(24.9%)	63(27.8%)	159(23.6%)	18(27.7%)	15(26.3%)	
VRT alone		475(46.4%)	102(44.9%)	313(46.4%)	27(41.5%)	33(57.9%)	0.279
Chemotherapy		211(20.6%)	50(22.0%)	143(21.2%)	14(21.5%)	4(7.0%)	0.035
Late rectal toxicity Grade≥2		40(3.9%)	6(2.6%)	25(3.7%)	3(5.4%)	6(10.4%)	0.08
Late urinary toxicity Grade≥2		7(0.7%)	0	6(0.8%)	1(1.8%)	0	0.399
Late hematologic toxicity Grade≥2		13(1.3%)	2(1.0%)	10(1.5%)	0	1(2.1%)	0.741
Cancer-specific mortality		38(3.7%)	4(1.8%)	20(3.0%)	5(7.7%)	9(15.8%)	0

**Figure 3:**
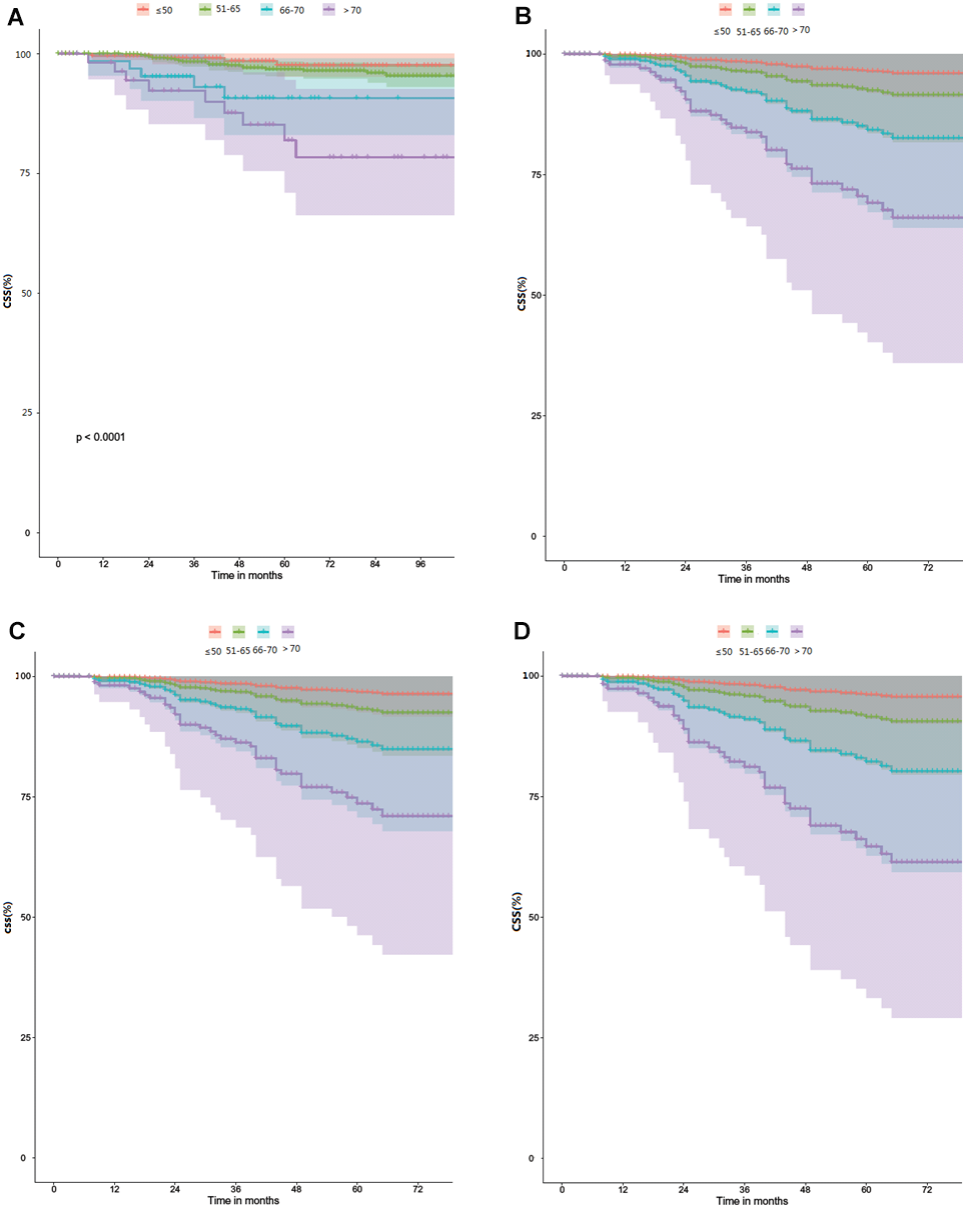
**Comparison of CSS between the different age groups.** (**A**) Univariate and unadjusted for age groups. (**B**) Adjusted for age groups, MI, LVSI, LUSI, CSI, Grade and Pathology. (**C**) Adjusted for the same variable as in B, plus additionally surgical type and EBRT±VBT. (**D**) Adjusted for the same variable as inC, plus additionally chemotherapy.

The survival analysis of each age group showed that the CSS of the oldest group was significantly lower than that of the younger group (P < 0.0001) (Figure 3A). After adjusting the covariates related to cancer characteristics, such as MI, LVSI, LUSI, CSI, grade and pathology, the difference in survival curves for each age group was more obvious (Figure 3B); after additional local treatment factors (surgical type and EBRT±VBT), the curve increased slightly(Figure 3C). After additional chemotherapy, CSS decreased significantly in the oldest group (Figure 3D). After adjusting for the above factors, the change of HRs in cancer-specific mortality at different age groups is shown in Table 5. The HRs of age on CSS in the patients >65y group were more than 4 times as high as that in the patients≤65y group in unadjusted model. After adjusting for the cancer characteristics, surgical type and EBRT±VBT covariates, the HR of age on CSS decreased from 5.516 to 4.618 and from 2.509 to 2.091 respectively for the patients over 70y group and the patients 66-70y group. But after adjusting for the above covariates plus additionally chemotherapy and treatment-related late toxicity covariates, the HR of age on CSS increased from 4.618 to 5.821 and decreased from 2.091 to 1.621 respectively for the patients over 70y group and the patients 66-70y group. There was no significant change in HRs between the two younger groups after adjustment.

**Table 5:** Univariable and multivariable HRs of EC cancer-specific death by the four age-at-diagnosis groups.

**Age at diagnosis**	**N**	**Events**	**5-year CSS**	**Model 1^a^**	**Model 2^b^**	**Model 3^c^**	**Model 4^d^**	**Model5^e^**	**Model6^f^**
				**HR (95% CI)**	**HR (95% CI)**	**HR (95% CI)**	**HR (95% CI)**	**HR (95% CI)**	**HR (95% CI)**
≤50	227	4	97.6%	0.407(0.144-1.146)	0.457(0.159-1.310)	0.428(0.149-1.229)	0.423(0.147-1.215)	0.421(0.146-1.210)	0.250(0.058-1.069)
51-65	675	20	96.8%	0.559(0.296-1.057)	0.546(0.286-1.043)	0.589(0.306-1.133)	0.591(0.307-1.136)	0.566(0.293-1.092)	0.705(0.333-1.491)
66-70	65	5	90.6%	2.509(0.978-6.433)	2.074 (0.801-5.373)	2.101(0.811-5.447)	2.091 (0.807-5.420)	2.157(0.832-5.592)	1.621(0.474-5.549)
>70	57	9	81.9%	5.516(2.61-11.66)	5.015(2.264-11.11)	4.469(1.980-10.09)	4.618(2.038-10.46)	5.334(2.324--12.24)	5.821(2.296-14.76)

^a^Model 1 Univariate and unadjusted for age groups. ^b^Model 2 Adjusted for age, MI, LVSI, LUSI, CSI, grade, and pathology. ^c^Model 3 Adjusted for the same variables as in Model 2, plus additionally surgical type. ^d^Model 4 Adjusted for the same variable as in Model 3, plus additionally EBRT±VBT. ^e^Model 5 Adjusted for the same variable as in Model 4, plus additionally chemotherapy. ^f^Model 6 Adjusted for the same variable as in Model 5, plus additionally treatment-related late toxicity.

## DISCUSSION

Data published in 2021 showed that the incidence rate of EC increased continuously in China in 2015, and the age of patients was younger, and most of them were in the early-stage [1]. In the comparison of the East and the West, age was negatively correlated with CSS. However, in the East, there were more patients with Grade 1 and 2, T1a and less patients with T2 in early-stage EC (P < 0.05) (see Supplementary Materials). Therefore, it is very important to study the clinical characteristics of early-stage EC in China.

Age is considered an important prognostic factor in patients with EC. In most of previous studies, age has been mostly used in multivariate analyses as a categorical variable, and the cut-off values for age ranged from 50-70y [5–13]. Dichotomy also simplifies statistical analysis and the interpretation and presentation of results. However, much prognosis information is lost because of the dichotomization of age. Therefore, this study mainly introduces age into the Cox model as a continuous variable to analyze the impact on the survival and treatment choices of patients with early-stage EC. The results show that not only older age but all age as a continuous variable was an independent prognostic factor of CSS, and the HR of cancer-specific mortality increased steadily with age.

Age is an important prognostic factor for EC. Although the age of the patients tends to be younger, the elderly patients are still the main group. Moreover, elderly patients with EC usually have more aggressive clinicopathological features, more conservative treatment and poor survival [12–16]. It was also observed in this study that older patients experienced more treatment-related toxicities. These indicated it’s possible that the worse outcome related to worse prognostic factors and the lack of appropriate adjuvant therapy for the older population. However, previous studies have not clearly put forward the prognostic value of age in each age group. Therefore, in order to clarify the prognostic role of age in different age groups, we conducted further analysis.

We divided all patients into four age groups in this study. In GOG-99 and PORTEC studies, age was emphasized as an important prognostic factor. In the same risk population, the older the age is, the less risk factors needed to be combined. In the two studies, the cut-off values of age were set as 50-70 years and 60 years respectively [10, 11]. In our study, the age cutoff point of 65 years was significant for CSS. Based on the above studies and our results, we divided age into four categories: ≤50 y, 51-65 y, 66-70 y and >70 y. The HRs of age on CSS were analyzed by univariate and multivariate statistics in our analysis.

On the whole, age was a protective factor for patients≤ 65 y and an independent poor prognostic factor for patients >65 y. In all age groups, no matter what factors are adjusted, HR still tended to increase with the increase of age.

In all age groups, adequate treatment did not change HRs for age. However, after considering the treatment-related toxicities, HR of patients >70 y increased the most, while HRs of other age groups were basically the same or even decreased. Therefore, for patients ≤ 70 y, they maybe benefit from adequate treatment. However, more attention should be paid to the treatment-related toxicities for patients over 70. Thus, it is recommended to consider fully the impact of treatment on quality of life. This perspective is consistent with the view of Armbruster SD et al [17].

Therefore, this study further verified that old age itself is an independent poor prognostic factor, independent of clinicopathological features and treatment methods. We need to pay more attention to the treatment-related toxicities. For very elderly patients, treatment-related toxicities may increase the risk of age.

Surgery is the primary choice for early-stage EC, and postoperative adjuvant treatment is given according to the risk factors. In this study, the surgical methods only focused on incompletely surgically staging and complete surgically staging, but did not focus on whether minimally invasive surgery and sentinel lymph node identification. Previous literature reported that the choice of different surgical methods, especially minimally invasive surgery or sentinel lymph node identification, can reduce the perioperative complications of patients and reduce the risk of death [18–20]. In NCCN Guidelines Version 1.2020 and the latest ESGO/ESTRO/ESP guidelines on EC [21], molecular typing was included in the risk classification of patients. In addition, Accumulating evidence suggests the potential use of novel biomarkers for EC prognosis to screen high-risk groups [22, 23]. Therefore, in the choice of therapeutic schedule, especially EBRT, BRT or chemotherapy, it is recommended to combine with clinicopathological factors, molecular typing and related biomarkers to clarify the risk classification, and give the elderly patients appropriate individualized treatment with lower toxicities.

One of the limitations of the current study is multicenter and retrospective, which leads to the lack of some surgical information and medical comorbidity data. For the elderly, medical comorbidity are also an important factor affecting the choice of therapeutic schedule [13, 24, 25]. In addition, due to the limitation of enrollment time, there is no molecular typing in the current data.

However, as we known, this is the largest amount of data available in the Chinese population on early stage EC post operation and adjuvant treatment. In this study, age was analyzed as a continuous variable and retained a large amount of prognostic information. In clinical practice, the results may provide a new insight for the management of early-stage EC.

## CONCLUSIONS

Age as a continuous variable is an independent prognostic factor and 65 year-old may be the best cut-off point for CSS in patients with early-stage EC in the Asian population. With an increase in age, the risk of cancer-specific mortality increases gradually. Age is an adverse prognostic factor for CSS in patients > 65y, but it is a protective factor for patients ≤65y. Quality of life should be given greater weight in the choice of therapeutic schedule for those patients >70 y.

## MATERIALS AND METHODS

### Ethics approval and informed consent

This retrospective study was approved by the Ethics Review Committee of Peking Union Medical College Hospital, Chinese Academy of Medical Sciences [Protocol number S-K139]. The clinical trial ID of the study is ChiCTR-PRC-17010712. Evaluation of all data met the requirements of the Helsinki Declaration.

### Materials and evaluation

After Institutional Review Board approval, we retrospectively reviewed the medical records of patients in our multi-institutional EC database. A total of 1024 patients with early–stage EC treated at 13 grade A tertiary hospitals in China between January 1999 and December 2015 were included. All patients underwent primary hysterectomy/bilateral salpingo-oophorectomy and adjuvant radiotherapy. All patients had FIGO 2009 stage I or stage II EC, World Health Organization performance score 0 to 2, and a minimum of 3 months of follow-up after adjuvant radiotherapy. Risk classification was carried out according to ESMO-ESGO-ESTRO risk classification. The treatment-related late toxicity was evaluated according to Radiation Therapy Oncology Group (RTOG) criteria.

### Radiotherapy modalities

All patients received postoperative pelvic external beam radiotherapy (EBRT) and vaginal brachytherapy (VBT) or VBT alone. The target volume of EBRT included the vaginal stump and upper 1/2 of the vagina, parauterine, presacral, obturator, internal and external iliac and common iliac lymphatic drainage areas. Conventional four field “box” radiotherapy (CRT), three-dimensional conformal radiotherapy (3DCRT) and intensity-modulated radiotherapy (IMRT) were used in external beam irradiation. VBT was performed by irradiating the vaginal stump and upper 1/2 of the vagina with single-channel or multichannel applicators, and two- and three-dimensional HDR brachytherapy plans were used for brachytherapy.

### Follow up

All patients had complete clinical, pathological, and follow-up information. Patients were assessed every 3-6 months for the first 2 years after radiotherapy, every 6-12 months during the following three years, and then annually. The prognostic factors analyzed included age at diagnosis, surgical type, pathological type, grade, myometrial invasion (MI), lymphatic vascular space invasion (LVSI), lower uterine segment invasion (LUSI), cervical stromal invasion (CSI), FIGO 2009 stage, ESMO-ESGO-ESTRO risk classification, time interval between surgery and radiotherapy, radiotherapy mode, and use of chemotherapy.

The primary endpoint was 5-year cancer-specific survival (CSS) and the secondary endpoint was treatment-related late toxicity. The endpoint events occurred from the beginning of radiotherapy to cancer-specific death or the last follow-up time. After the initial treatment, the new lesions in the pelvic area were defined as local regional recurrence (including vaginal recurrence), and the new lesions beyond the pelvic area were defined as distant metastasis. The Kaplan-Meier method was used to calculate the cancer-specific survival (CSS).

### Statistical analysis methods

The risk ratio (HR) and 95% confidence interval (CI) were calculated by Cox proportional hazards regression. Age as a continuous variable was used in the multivariate analysis. In the stratified analysis by age, the receiver operating characteristic curve (ROC) was used to obtain the best cut-off value. Age after stratification was divided into four age groups (≤50 y, 51-65 y, 66-70 y, and > 70 y). The changes in HRs in each group were observed by running the unadjusted model and the adjusted model for cancer characteristics, treatment modalities and treatment-related late toxicity. R version 4.0.2 produced by R Core Team was used for statistical analysis. All P values were two sided, and P < 0.05 was considered significant.

## Supplementary Material

Supplementary Materials
