# Supplementary material for: Effect of age as a continuous variable in early-stage endometrial carcinoma: a multi-institutional analysis in China
Source: Aging (Albany NY). 2021 Aug 9;13(15):19561–74. doi: 10.18632/aging.203367 (PMC8386535; doi:10.18632/aging.203367)
Supplement: Supplementary Materials [file aging-13-203367-s001.pdf]

SUPPLEMENTARY MATERIALS

We searched the SEER database from 1999 to 2015 for patients with uterine tumor who had received surgery and radiotherapy, and got a total of 188644 patients. We screened the overall data. We excluded 17650 cases of sarcoma, 30501 cases of unknown grade, 117381 cases who received particle, isotope, refused radiotherapy or unknown, 18160 cases of T0/TX/T3-4 or blank, 1322 cases of N1-2 or Nx, 432 cases of non postoperative radiotherapy, 418 cases of unknown cause of death and 271 cases of unknown race. Finally, a total of 2509 patients were included in the analysis.

To quantify the prognostic effect of age at diagnosis, the patient's age was also used as a continuous variable, and P-spline was used to enter Cox proportional hazards regression in smooth HR. The results showed that the risk (lnHR) of mortality increased steadily with age (Supplementary Figure 1). In the Figure 1, 67 years old was the cut-off value of 2509 patients calculated by ROC. The spearman correlation analysis of 2509 patients in SEER database showed that age was negatively correlated with cancer-specific survival (CSS) (P=0.002). This result is consistent with our data of 1024 patients with early-staged endometrial carcinoma in China.

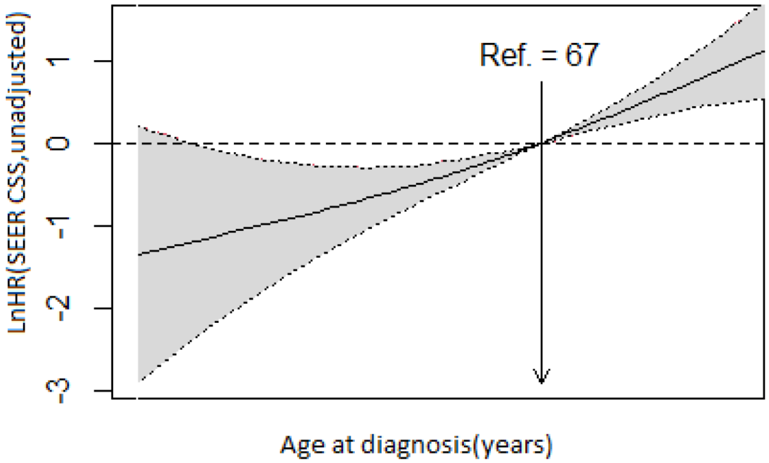

Supplementary Figure 1. Linear-dependent effect of increasing age on CSS. The estimated logarithm HRs (lnHR, solid line) with 95% CIs (shading) for the association of patient age at diagnosis with CSS in 2509 patients based on the *dfmacox*as smooth HR – the optimal extended Cox-type additive hazard regression unadjusted model. *dfmacox*=degrees of freedom in multivariate additive Cox models.

Considering the survival difference of different races, we further compared the CSS difference between white and black in 2509 patients from SEER database. In the comparison of the White and the Black, it was found that there were more Grade 1, Grade 2 in the White with early-staged EC ($P < 0.05$) (Supplementary Table 1). The 5-year CSS of the White and the Black were 84.8% and 75% respectively (X2=6.519, P = 0.011) (Supplementary Figure 2). This survival difference may be associated with more well differentiated cases in the White.

Supplementary Table 1. Comparison of clinical characteristics between the White and Black.

Characteristic		All patients (%)		X2	P
		White(2227)	Black(282)		
Grade	1	486(21.8%)	35(12.4%)	45.2	0
	2	863(38.8%)	89(31.6%)		
	3	695(31.2%)	106(37.6%)		
	4	183(8.2%)	52(18.4%)		
Stage	T1a	561(25.2%)	109(38.6%)	31.2	0
	T1b	1009(45.3%)	84(29.8%)		
	T2	657(29.5%)	89(31.6%)		

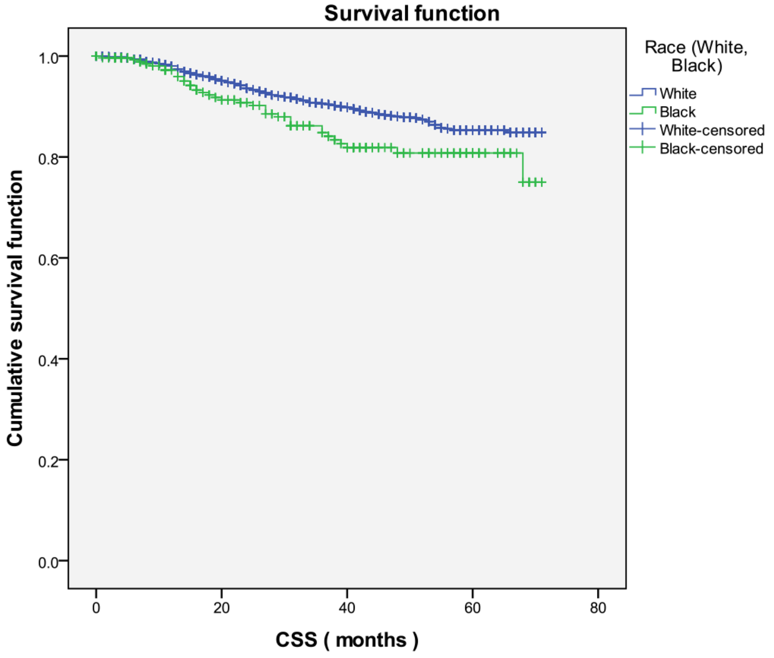

Supplementary Figure 2. Cancer-specific survival between the white and black. We compared 1024 Chinese patients in this study with 2509 Western patients from SEER database. In the comparison of the East and the West, it was found that there were more Grade 1, Grade 2, stage T1a and less stage T2 in the Chinese patients with early-staged EC (P < 0.05) (Table 2). The 5-year CSS of the East and the West were 95.7% and 84.4% respectively (X2=67.2, P=0) (Figure 3). This survival difference may be related to the different risk classification of patients. In our data, there were fewer clinicopathological risk factors and earlier stage.

We compared 1024 Chinese patients in this study with 2509 Western patients from SEER database. In the comparison of the East and the West, it was found that there were more Grade 1, Grade 2, stage T1a and less stage T2 in the Chinese patients with early-staged EC (P < 0.05) (Supplementary Table 2). The 5-year CSS of the East and the West were 95.7% and 84.4% respectively (X2=67.2, P=0) (Supplementary Figure 3). This survival difference may be related to the different risk classification of patients. In our data, there were fewer clinicopathological risk factors and earlier stage.

Supplementary Table 2. Comparison of clinical characteristics between the East and West.

Characteristic		All patients (%)		X2	P
		East(1204)	West(2509)		
Grade	1	334(32.6%)	521(20.8%)	107.4	0
	2	444(43.3%)	952(37.9%)		
	3	188(18.4%)	801(31.9%)		
	4	58(5.7%)	235(9.4%)		
Stage	T1a	474(46.3%)	670(26.7%)	173.4	0
	T1b	421(41.4%)	1093(43.6%)		
	T2	129(12.6%)	746(29.7%)		

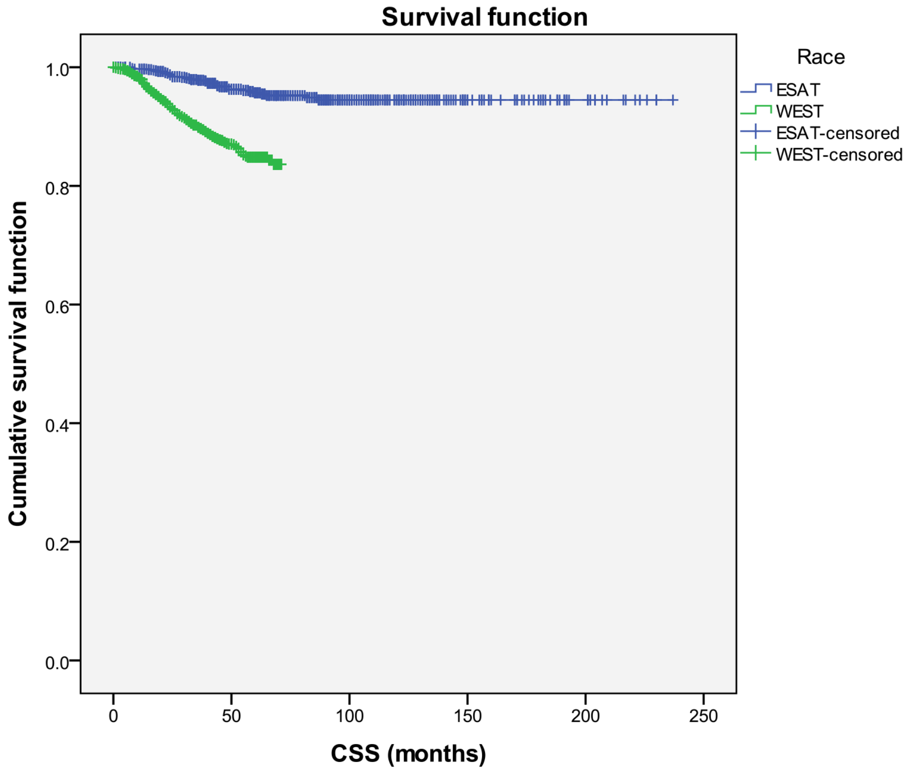

Supplementary Figure 3. Cancer-specific survival between the east and west.
